# Breast Cancer Surgery: New Issues

**DOI:** 10.3390/curroncol28050344

**Published:** 2021-10-11

**Authors:** Francesca Magnoni, Sofia Alessandrini, Luca Alberti, Andrea Polizzi, Anna Rotili, Paolo Veronesi, Giovanni Corso

**Affiliations:** 1 Division of Breast Surgery, IEO European Institute of Oncology, IRCCS, 20141 Milan, Italy; sofia.alessandrini@ieo.it (S.A.); luca.alberti@ieo.it (L.A.); andrea.polizzi@ieo.it (A.P.); paolo.veronesi@ieo.it (P.V.); giovanni.corso@ieo.it (G.C.); 2 Division of Breast Radiology, IEO European Institute of Oncology, IRCCS, 20141 Milan, Italy; anna.rotili@ieo.it; 3 Department of Oncology and Hemato-Oncology, University of Milan, 20122 Milan, Italy

**Keywords:** breast cancer, breast cancer surgery, breast conservative surgery, nipple sparing mastectomy, neo-adjuvant chemotherapy, hereditary breast cancer, margin

## Abstract

Since ancient times, breast cancer treatment has crucially relied on surgeons and clinicians making great efforts to find increasingly conservative approaches to cure the tumor. In the Halstedian era (mid-late 19th century), the predominant practice consisted of the radical and disfiguring removal of the breast, much to the detriment of women’s psycho-physical well-being. Thanks to enlightened scientists such as Professor Umberto Veronesi, breast cancer surgery has since impressively progressed and adopted a much more conservative approach. Over the last three decades, a better understanding of tumor biology and of its significant biomarkers has made the assessment of genetic and molecular profiles increasingly important. At the same time, neo-adjuvant treatments have been introduced, and great improvements in genetics, imaging technologies and in both oncological and reconstructive surgical techniques have been made. The future of breast cancer management must now rest on an ever more precise and targeted type of surgery that, through an increasingly multidisciplinary and personalized approach, can ensure oncological radicality while offering the best possible quality of life.

## 1. Introduction

**Figure 1:**
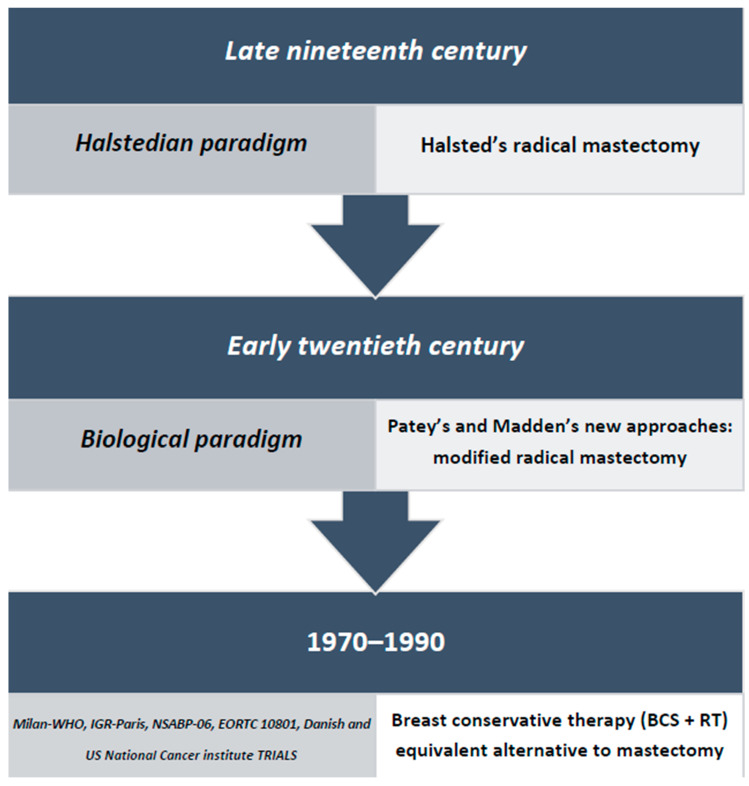
Evolution of breast cancer (BC) surgery.

It is known that mastectomy has been the treatment of choice for breast cancer (BC) since the times of the Byzantine Empire [1,2]. Fast-forwarding to the modern era, the Scottish surgeon Benjamin Bell (1749–1806), despite being a precursor of skin conservation in the mastectomy technique, “advised that the whole breast be removed even if the lump was small” [3], perfectly exemplifying the dominant scientific approach to the surgical treatment of breast cancer in those times. In the first half of the 19th century, little further progress in breast cancer surgical techniques was achieved. Indeed, the shared belief was that BC required the extirpation of the entire organ and of its contiguous anatomical structures: “the story was not one of ordinary progression, but fraught with retrogressions…The horror of sepsis, the need for anesthesia, and the wide acceptance of the incurability of cancer were prominent in delaying the development of surgery of the breast” [4]. In the second part of the 19th century, thanks to advances in anesthesia and antisepsis, William Stewart Halsted (1852–1922) validated a technique previously studied and promoted by a number of other surgeons [4] and that consisted in the en bloc resection of the breast, i.e., removal of the breast, pectoral muscles and ipsilateral axillary nodes. Based on Virchow’s theory of the centrifugal dissemination of BC (BC being considered to be a localized disease at inception), the so called “Halstedian paradigm” (Figure 1) was further corroborated by the observation that local recurrence rates had dramatically decreased following the application of the Halstedian technique. Halsted himself reported a 6% local recurrence rate at 3 years, while his European counterparts registered local recurrence rates ranging from 50 to 80% [4,5]. Moreover, in his several scientific reports to the American Surgical Association, Halsted pointed out the importance of early diagnosis in improving breast cancer prognosis, together with the possibility of performing minor axillary dissections, which lacked any real benefit on survival [4]. A new standard in the development of breast cancer surgery emerged that was defined by the three elements on which Halsted had based his work: the concept of cancer dissemination, a meticulous operating technique and a logically and scientifically sound approach [4]. Halsted basic assumption was that “more is better”: the more extensive the breast cancer local excision was, the greater the chance of survival and of minimizing local recurrences. In Halsted’s time, breast cancer was often diagnosed at an advanced stage. Therefore, although the clinical stage of presentation of the disease was being taken into consideration, Halsted’s technique had many adverse effects due to it being very destructive in terms of both consequent physical disability and psycho-sexual suffering. Nonetheless, it became established as the primary surgical treatment for all operable breast cancers and continued to prevail until the middle of the 20th century [6]. In the second half of the 19th century, however, slow scientific progresses in radiotherapy, chemotherapy, hormonal therapy, pathology and the improvement of the cancer staging system greatly contributed to a better understanding of the disease and improved breast cancer patients’ management, challenging the central role of Halsted’s radical mastectomy. Since then, the improvement of the physical and psychological well-being of women has been the main focus in breast cancer surgery development with priority given to increasingly less disfiguring and radical approaches of oncological surgery.

## 2. “Less Is More, Bigger Is Not Better”

Since the beginning of the 1900s, the belief that “less is more, bigger is not better”—a belief whose meaning is crystal clear from the modern scientific perspective on breast cancer surgical management [7,8]—has been propelling the evolution of breast cancer surgery towards an increasingly conservative approach.

In the first half of the 20th century, radical mastectomy gradually became the object of intense international scrutiny. Most notably, its routine use was questioned by the English surgeon David H. Patey (1899–1977), the first to modify Halsted’s approach by not removing the great pectoral muscle [9], and by his colleague John Madden (1912–1999), who described the same procedure used by Patey. By completely removing the axillary nodes while leaving both pectoral muscles in place, this modified radical mastectomy represented a less disfiguring procedure with still reduced post-operative morbidity rate [10]. Gradually, it became more and more frequently used and, finally, emerged as the standard of care for breast cancer patients [6]. The growing importance of early diagnosis and the gradual introduction of mammography as its main tool, alongside the hypothesis that breast cancer has a systemic biological potential, inspired six widely known, large trials, which came to the same game-changing conclusion: in women with stage I and II breast cancer, breast-conserving surgery (BCS) followed by post-operative radiotherapy (RT), despite being associated with higher rates of local recurrence, does not affect the overall and the disease-free survival rates seen with mastectomy. The six trials in question were: the Milan World Health Organization (WHO), the Institute Gustave–Roussy (IGR-Paris), the National Surgical Adjuvant Breast and Bowel Project (NSABP)-06, the European Organization for the Research and Treatment of Cancer (EORTC) 10801, the Danish and the U.S. National Cancer Institute trial [11,12,13,14,15,16]. In 1990, the U.S. National Institutes of Health (NIH) held a Consensus Development Conference [17] that reviewed the results of these randomized prospective trials and declared that “breast conservation therapy is an appropriate method of primary therapy for the majority of women with stage I and stage II breast cancer and is preferable because it provides survival equivalent to total mastectomy and axillary dissection while preserving the breast.” [18]. Long-term follow-up results of these key trials [19,20,21,22,23,24] have confirmed that BCS with postoperative RT has, compared to mastectomy, similar effects on mortality even if it is associated with an increased risk of locoregional recurrence. This was found to be especially true in the National Cancer Institute trial, with local recurrence in 19% of BCS with RT, versus 6% of mastectomy (*p* = 0.01), and in the EORTC trial, with a 20% local recurrence rate for BCS with RT vs. 12% for mastectomy (*p* = 0.01) [20,25]. One of these studies was conducted from 1973 to 1980 by Professor Umberto Veronesi. It included 701 patients with invasive breast cancer <2 cm in diameter, with clinically negative lymph nodes, who were randomly subjected to either radical mastectomy or quadrantectomy and axillary dissection, followed by radiotherapy (QUART). Local recurrences and survival rates were found to be the same in the two cases [26]. Professor Veronesi was an early pioneer of this modern surgical approach, as documented by the speech he gave at the World Health Organization in Geneva in 1969: “Some years ago, I purposed, during a scientific meeting, to compare the traditional mutilating mastectomy with a new conservative surgical approach (the so-called quadrantectomy). At this time, I sensed that the radical Halsted mastectomy was not always necessary. This was not well received by the audience, and they thought I was a crazy physician” [27]. Over the last two decades of the 20th century, results of the I, II and III Milan trials, together with those from analogous studies, have consolidated the breast-conserving approach in the clinical practice. The Veronesi quadrantectomy represented a great advance in breast cancer surgery as it exemplified the new concept of a “minimum effective treatment” [27] that would adequately control the disease while achieving excellent cosmetic results. Until then, breast cancer had been considered a predominantly systemic disease. The Veronesi quadrantectomy came to embody a new alternative view that was emerging in those years and that was based on the novel paradigm of biological pre-determinism (Figure 1), according to which overall survival does not depend on the extent of the primary surgery but is pre-determined by the micrometastases present at the time of diagnosis [28,29].

The increased rate of local recurrence observed in the trials could be ascribed to several causes, including an inadequate patient selection, an inappropriately performed surgery or radiotherapy or a biologically aggressive disease [30]. BC patients’ accurate selection and proper treatment allowed them to safely choose breast conservation over mastectomy, as proper management avoids any increase in the risk of local failure [30]. Indeed, a meta-analysis of nine prospective randomized trials comparing conservative surgery followed by radiation with mastectomy has found no difference in survival rates [31]. Nonetheless, recent meta-analyses [32] have reinforced the link between local control and survival, pointing out the importance of locoregional therapies. To date, significant improvements in pre-operative assessment, in genetic predisposition evaluation and in targeted sub-type specific systemic therapies, have greatly reduced the rates of locoregional recurrence since the first randomized trials [7]. In the NSABP B-06 trial, conducted in the 1970s, the 20-year ipsilateral recurrence rate was 14.3% [19]. In the NSABP trials conducted in the 1990s, the 10-year local recurrence rate among patients treated with breast-conserving surgery ranged from 3.5% to 6.5% instead [33].

## 3. Margin Status in Breast-Conserving Surgery and Molecular Subtypes Implication

The selection criteria for breast-conserving therapy (BCT, i.e., BCS followed by RT) are strictly dependent on the following factors: multicentricity, extent of the calcifications in the areas adjacent to the tumor, expertise in safely releasing radiotherapy, patient preference and, last but not least, extent of the disease in the breast, given a suitable ratio of breast size to tumor size and, therefore, margins status [34].

An Italian Network of Senology Centers, Senonetwork Italia, recommends that specific techniques should be applied and preoperative procedures followed to obtain negative margins and reduce the probability of a second operation, especially in case of occult cancers [34]. Based on the cases of impalpable and clinically occult lesions treated at one single institute, procedures such as charcoal, metal wire and radio-guided occult lesion localization (ROLL) can help accurately identify the cancer site [35].

The 2019 St Gallen International Consensus Guidelines state: “No ink on tumor is a sufficient surgical margin in most cases of primary invasive breast cancer, including patients with lobular breast cancer or extensive intraductal components, and after resection of residual palpable or imaging abnormalities following neoadjuvant systemic therapy” [36]. This recommendation applies to a number of wide-ranging contexts. Indeed, the implications a negative margin width might have on local recurrence have been the subject of several studies, which have in turn generated different guidelines and failed to reach any unanimous consensus when concluding that the adequate width for a negative margin can range from no ink on tumor to 5 mm or greater [37].

The oncoplastic approach has improved the chances to obtain better cosmetic results after BCS, in particular in patients with a less favorable ratio between breast size and tumor size, with large breasts or with a tumor sited in challenging quadrants, such as the central or the inferior [28].

Despite wider margins now being achievable thanks to oncoplastic techniques, a univocal definition of a histologically negative margin for either invasive or in situ disease has been lacking for years. Consequently, the number of patients opting for mastectomy has been steadily increasing despite the suggested oncological equivalence in survival of the two approaches in case of early BC [38]. 

Within this vague scenario—aptly described by Monica Morrow as “Margins in breast-conserving therapy: have we lost sight of the big picture?” in the title of her 2008 editorial [39]—the acknowledgement of the substantial additional benefits of long-term local control of tumor biology, rather than of tumor burden [33,40,41], has led the Society of Surgical Oncology (SSO) and the American Society for Radiation Oncology (ASTRO) to establish, in 2014, evidence-based consensus guidelines on margins for BCS of early-stage invasive breast cancer [42]. The no-ink-on-tumor definition of a negative margin has led to the standardization of the surgical approach in BCS by reducing unnecessary reoperation rates to achieve widely clear margins, either with margin re-excision or conversion to mastectomy [43]. A consensus on careful histological assessment of resection margins has also led to recommending tumor preferably >2 mm in case of in situ disease [38,44].

**Figure 2:**
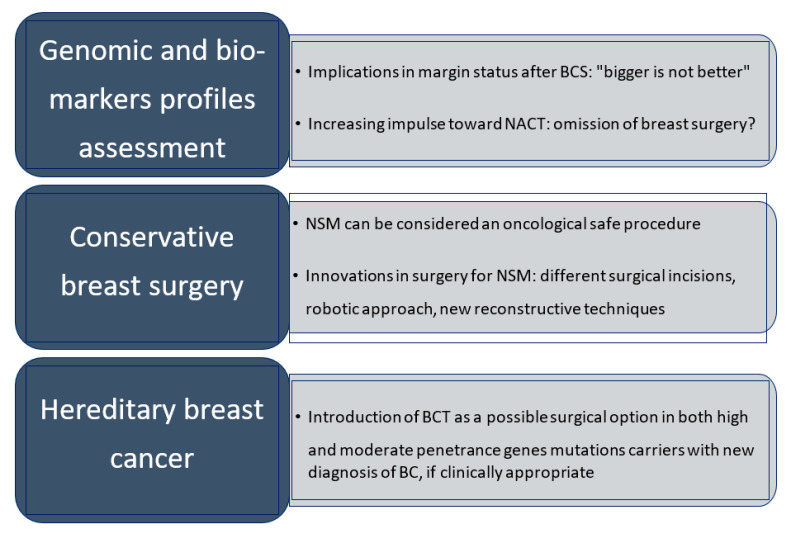
Last three decades’ advances in de-escalating BC surgery. BCS: breast conservative surgery; NACT: neo-adjuvant chemotherapy; NSM: nipple sparing mastectomy; BC: breast cancer.

In this context, the determination of the BC molecular subtype has recently emerged as a new effective tool in invasive BC management and local control. Adjuvant systemic therapy that reduces the risk of distant metastasis also appears to reduce local recurrence [40] (Figure 2). In the National Surgical Adjuvant Breast and Bowel Project (NSABP) Protocol B14, local recurrence was described in 14.7% of the estrogen receptor positive patients that received placebo compared to only 4.3% of those treated with tamoxifen [45]. Likewise, in the NSABP B13, which compared methotrexate and five fluorouracil chemotherapy to no treatment in estrogen receptor negative patients, the incidence of local recurrence was reduced from 13.4% to 2.6% with chemotherapy [46]. Moreover, the influence of tumor biology on local recurrence became evident when the possibility of predicting local and regional recurrence from the 21-gene recurrence score assay (Oncotype DX) was reported [47].

Evidence has, thus, shown that breast cancer actually consists of a series of genetically distinct diseases each with a different prognosis. Biological factors, such as tumor histology and grade, estrogen receptor, progesterone receptor and HER2 status, must now be taken into consideration when choosing between BCT and mastectomy [41,48]. The perspective on early BC is currently evolving. Undoubtedly, BCS is recommended for patients with early BC, due to the oncological equivalence of BCS followed by RT and mastectomy [38]. However, recent population-based studies have reported improved overall survival after BCS plus RT, compared to mastectomy, demonstrating that BCT has significant benefits, even independently of measured confounders [49,50,51]. Indeed, concerns about the surgical oncological equivalence of mastectomy and BCS have emerged and become a starting point for further studies [51].

## 4. Second Breast Conservative Surgery for Ipsilateral Breast Tumor Recurrence: The New Standard of Treatment

The surgical treatment of an ipsilateral breast tumor recurrence (IBCR) is influenced by the tumor’s biological and pathological traits. Houvenaeghel and colleagues, for instance, reported a shorter disease-free interval in young patients with high-grade BC of the aggressive molecular subtype [52]. Furthermore, Corso et al. observed that metastatic axillary lymph nodes (*p* = 0.0004), high-grade (G3) (*p* = 0.04), HER-2 positive and triple negative tumors (*p* = 0.008, *p* = 0.02, respectively) were significantly associated with an increased risk of IBCR and, instead, highlighted the protective role of adjuvant treatments [53]. Subsequently, the same authors pointed out the importance of novel validated nomograms in predicting the risk of IBCR in patients treated with either BCS or mastectomy [54].

The management of ipsilateral breast tumor recurrence (IBTR) after BCT remains a matter of debate. The National Comprehensive Cancer Network (NCCN) guidelines currently recommend performing mastectomy according to the established practice, mainly due to issues related to re-irradiation [55]. However, a not so small number of publications has provided evidence in support of the oncological safety of a BCS repetition in the event of ipsilateral recurrence, also associated with re-irradiation [56,57,58,59,60,61]. 

Similarly, some of these studies have shown the importance of operating a selection amongst BC patients to identify those suitable for a second BCS. Gentilini and colleagues, for example, suggested that the best candidates for re-conservation are the patients with small tumors and late IBTRs [58]. In a recent retrospective study by Sagona and colleagues, age < 65 years (*p* = 0.018) and disease-free interval < 24 months (*p* = 0.007) were found to significantly increase the probability of recurring to mastectomy. The authors concluded by supporting a second BCS for IBCR, due to the acceptable locoregional control and survival [60], as further corroborated by later analyses of propensity score matching [61]. 

To date, re-conservation has proven increasingly popular and was recently found to have grown from 27% between 2000 and 2004 to 61% between 2015 and 2019 [62].

Currently, there is no phase III prospective randomized trial comparing these two treatment options [62]. Recently, the GEC-ESTRO breast cancer working group used a propensity score-matched cohort analysis to compare oncological outcomes between 377 patients subjected to second BCS and 377 treated with salvage mastectomy. This robust statistical method analysis demonstrated that, with a median follow-up of 6.3 years, oncological outcome results were not significantly different between the two cohorts in terms of disease-free survival (82.5 vs. 78.6%), cause-specific survival (91.2 vs. 91.8%) or overall survival (86.7 vs. 87.5%) [63]. Subsequent data did not show any significant differences at 10-year follow-up either [63].

Again, a recent single institution study conducted at the Memorial Sloan Kettering Cancer Center retrospectively compared the oncological outcome of treating isolated IBTR following breast conservation surgery (322 pts) either with breast re-conservation (40%) or with salvage mastectomy (60%) [62]. Older age, long disease free-interval, lack of radiotherapy and use of the endocrine therapy as the initial adjuvant treatment were the clinical elements that significantly correlated with higher rates of re-conservations options [62]. 

Hence, current data support the concept that mastectomy might not be the only salvage choice for IBTR. Re-conservation should also include BCS and re-irradiation of the tumor bed and be discussed as an alternative to mastectomy after multidisciplinary evaluation [64] (Figure 2). The 2021 St Gallen Consensus Conference further confirmed the role of re-irradiation compared to mastectomy in case of IBTR after 5 years from BCT, especially in selected classes of patients [65].

## 5. Neo-Adjuvant Treatments: The Challenge of Nonoperative Management

Over the past two decades, systemic therapy with neo-adjuvant intent has been playing a rapidly growing role. Indeed, it has been proven to be a valid tool in downstaging both breast and axillary disease [66], not only in case of locally advanced BC, but also in selected cases of early-stage BC with biologically aggressive subtypes, such as triple-negative breast cancer and HER2-positive disease, that would normally require adjuvant chemotherapy [67]. Novel treatment regimens showed pathological complete response (pCR) rates of up to 70% and 30–40% for HER2-positive and triple-negative breast cancers, respectively [68,69,70]. These data might suggest reducing the extent of surgery for both the breast and the axilla after NACT. Indeed, the effects of sentinel node biopsy after NACT in cN1-2 downstaged cN0 have been widely studied and found to be associated with very positive results [71]. Consequently, the need for surgery of the primary tumor in the breast of patients defined “responders” to NACT has been questioned [72] (Figure 2).

Based on this assumption and thanks to the improved sensitivity and specificity reached by imaging and biopsy techniques, researchers are now trying to evaluate the oncological validity of omitting breast surgery for that selected subgroup of patients whose clinical complete response, defined before definitive surgery, and pCR, evaluated after surgery at the final histological report, are largely compatible [67,73,74]. A sign of things to come can be seen in three multicenter randomized trials, RESPONDER in Germany, Minimally Invasive Complete Response Assessment (MICRA) in the Netherlands and NOSTRA in the UK [74,75,76], which are evaluating the initial feasibility and reliability of pCR determination using minimally invasive image-guided biopsies of the tumor bed, instead of open surgery. Furthermore, the MD Anderson Cancer Center trial is looking at the effects on overall survival and ipsilateral breast tumor recurrence-free survival when, in cT1-2/cN0-1 BC patients who do not show any residual disease in image-guided biopsy after NACT, surgery is replaced with radiotherapy alone [28,77].

Experts have differing opinions on this matter [78]. Some of them have highlighted the importance of those imaging-guided biopsy data that gave discouraging false-negative rates ranging from 5% to 49%. Others have emphasized the promising results that vacuum-assisted biopsies have shown in pilot trials (false false-negative rate of about 5%) and drawn attention to new emerging technologies able to assess residual cancer with improved specificity. A prime example of them is the so-called intelligent vacuum-assisted biopsy, a modern machine capable of learning algorithms and assimilating the complex and heterogeneous interactions between vacuum-assisted biopsy, patient anamnesis, imaging and tumor biological variables [78,79].

At the European Institute of Oncology, a pilot study, is currently underway that aims at comparing the diagnostic accuracy of the histological results from vacuum-assisted biopsy performed before surgery, with final pathologic reports in patients submitted to NACT for triple-negative or HER2-positive ductal invasive BC, in case of pCR documented at imaging (mammography, ultrasound, magnetic resonance imaging (MRI) and positron emission tomography/computed tomography (PET/CT) scan). 

## 6. Conservative Mastectomy

Professor Umberto Veronesi’s motto “from maximum tolerable treatment to minimum effective treatment” reflects his life-time commitment to an ever increasingly conservative breast surgical approach [80]. A big step forward from the modified-radical mastectomy, both the skin-sparing mastectomy (SSM) and the nipple-sparing mastectomy (NSM), the so-called “conservative” mastectomies [80], with immediate breast reconstruction, embodied a new attitude in the field of BC treatment, characterized by a greater attention to women’s body image and well-being. In comparison to modified radical mastectomy, SSM has been universally recognized to have oncological validity in terms of survival and local recurrence [81,82,83,84]. NSM, instead, has raised major concerns in particular due to the small amount of ductal tissue behind the nipple–areola complex, considered to be at risk of local recurrence [85,86]. The reported rate of nipple–areola complex (NAC) involvement in NSM for BC ranges from 8 to 33% [86]. Consequently, in routine practice, performing a frozen section of retro-areolar tissue in therapeutic NSM is mandatory. Currently, the US National Comprehensive Cancer Network (NCCN) recommends NSM in those patients who meet specific clinical criteria, such as early stage, biologically favorable features, absence of nipple discharge and Paget’s disease [55]. The use of NSM in both therapeutic and prophylactic settings has been increasing significantly [86]. Indeed, several retrospective studies have demonstrated the oncological safety of NSM, reporting excellent outcome results and a very low local recurrence rate [86,87,88]. New guidelines aim at expanding the clinical applications of NSM, such as in the case of previous NACT [89] or of BC located close to the nipple [90]. 

Indeed, Wu and colleagues [89] performed a total of 1226 therapeutic NSM procedures and selected from them 319 NSMs in 310 patients after neoadjuvant chemotherapy. Over a mean follow-up of 63 ± 22 months, 38 cases presented LRR, six of which had NAC recurrence. The 5-year cumulative loco-regional recurrence and NAC recurrence rates were 11.0% and 1.9%, respectively. The 5-year OS was 91.3% in the whole population studied. Interestingly, at multivariate analysis, post-NACT Ki67 > 10% (hazard ratio, 4.245; 95% confidence interval, 1.865–9.663; *p* = 0.001) was found to be an independent risk factor for loco-regional recurrence and the only significant risk factor for NAC recurrence. In their study on 251 NSMs, Kim et al. [90] described that the tumor-to-nipple distance was ≤2 cm in 47.4% and ≤1 cm in 27.5% of cases. Over a mean follow-up period of 68.0 months, they also described a loco-regional recurrence rate of 4.4% and an overall survival rate at 5 years of 98.0% in patients with invasive cancer and of 100% in patients with “in situ” disease.

The increasingly conservative character of mastectomy is well represented in a recent report by Corso and co-workers, who documented the oncological and cosmetic effects of different types of surgical incisions during NMS, depending on the size of breast and areola and on the degree of breast ptosis [91] (Figure 2). The authors retrospectively investigated 117 surgical procedures performed in 100 patients with BC, specifically looking at the following types of incision: hemi-peri-areolar, round block approach or complete peri-areolar, vertical pattern and wise pattern. No significant correlations between clinical and pathological data, complications, pre- and post- surgery satisfactions and specific characteristics, such as ptosis and breast size, were identified, confirming that these techniques are oncologically safe [91].

In a recent retrospective study on 387 cases of NSM after previous breast surgery, the five-year overall survival and disease-free survival rates were 99.1 and 93.8%, respectively, with no nipple recurrence, thus supporting the safety of this procedure [92].

Technical innovations in reconstructive breast surgery have had a positive impact on cosmetic outcomes and women’s quality of life. The introduction of acellular dermal matrix and synthetic meshes and the pre-pectoral breast reconstruction offered to selected patients [93] are currently the subject of targeted randomized clinical trials aiming to evaluate their effects and increasingly extend their applicability in the future.

In Europe and Asia, robotic NSM has emerged as the novel approach in BC treatment and risk reduction and an alternative to the standard open technique [94,95,96,97]. Its safety and feasibility have been the subject of several studies [98]. A very recent phase III randomized controlled trial has concluded that robotic NSM is a safe procedure to perform given that its complications are similar to those of the traditional technique and that no local events have been observed at a median follow-up of 28.6 months [99]. The role of the minimally invasive technique in improving BC patients’ quality of life and in minimizing post-operative complications is also analyzed in a recent comparative study by Chen and colleagues, who have reported a decreased incidence of lymphedema after the Da Vinci Robot-assisted axillary lymph-node dissection during NSM [100].

## 7. Hereditary Breast Cancer and De-Escalation in Breast Surgery

The growing availability of genetic testing has led to prophylactic bilateral mastectomy becoming more common. Indeed, while presenting no difference in survival after BCT, women with BRCA mutations have been found to have an increased risk of new primary breast cancer and, therefore, to preferentially choose risk-reducing mastectomy surgery [101].

Currently, the managing of BC patients with ascertained genetic susceptibility for BC is evolving towards personalization. The evidence in support of different treatment options is good in the case of subjects with BRCA1/2 mutations but is rather limited for individuals with moderate penetrance mutations (such as those in the PALB2, CHEK2 and ATM genes) [102]. Besides, while guidelines on risk management abound, those on the role of local or systemic treatment in women with hereditary breast cancer are scarce [102]. Indeed, the debate on what the optimal local therapy for women with BRCA-associated breast carcinoma should be is still ongoing. 

As no randomized controlled trial has yet directly compared BCS and mastectomy for BRCA mutation carriers, the American Society of Clinical Oncology (ASCO), the American Society for Radiation Oncology (ASTRO) and the Society of Surgical Oncology (SSO) have provided guidelines on the management of BC in patients with germline mutations in the BRCA 1/2, PALB 2, CHEK 2 or ATM gene [101,102].

Nipple-sparing mastectomy is a suitable oncologic treatment and prophylactic option [101,103]. However, BCT should not be excluded from multidisciplinary discussion but should be offered to patients with newly diagnosed hereditary breast cancer, due also to the possibility of including RT, except in case of the TP53 mutation option (Figure 2) [101,103]. 

Recent studies have reported an association between CDH1 germline mutations and lobular breast cancer. In the context of the so-called hereditary lobular breast cancer, cancer risk management requires prophylactic mastectomy in case of an important family history of breast cancer aggregation [104].

## 8. Conclusions

Looking back at how the science of breast surgery has evolved, the advancements made in BC management, from the Halstedian to the modern era, appear very impressive and highly significant. It is not just the technical approach to breast surgery, with the preservation of gradually larger anatomical portions, that has evolved. Great advancements in genetics, molecular biology and imaging have also played a crucial role in improving outcomes as well as quality of life, psychology and physical image of the women affected. Nowadays, genomic profiling, molecular classification and novel diagnostic tools allow us to tailor breast cancer prevention and therapy to the single individual, in a new approach where multimodality and personalization of BC care have become paramount. Indeed, as the enlightened Dr. William J. Mayo would say, “the glory of Medicine is that is constantly moving forward, that there is always more to learn” [4].
